# EFR3 and phosphatidylinositol 4-kinase IIIα regulate insulin-stimulated glucose transport and GLUT4 dispersal in 3T3-L1 adipocytes

**DOI:** 10.1042/BSR20221181

**Published:** 2022-07-08

**Authors:** Anna M. Koester, Angéline Geiser, Kamilla M.E. Laidlaw, Silke Morris, Marie F.A. Cutiongco, Laura Stirrat, Nikolaj Gadegaard, Eckhard Boles, Hannah L. Black, Nia J. Bryant, Gwyn W. Gould

**Affiliations:** 1 Institute for Molecular, Cellular and Systems Biology, University of Glasgow, U.K.; 2 Strathclyde Institute of Pharmacy and Biomedical Sciences, University of Strathclyde, Glasgow, U.K.; 3 James Watt School of Engineering, University of Glasgow, U.K.; 4 Institute for Molecular Biosciences, Goethe-University 60438 Frankfurt, Germany; 5 Department of Biology and York Biomedical Research Institute, University of York, York, U.K.

**Keywords:** adipocytes, glucose transport, GLUT4, insulin

## Abstract

Insulin stimulates glucose transport in muscle and adipocytes. This is achieved by regulated delivery of intracellular glucose transporter (GLUT4)-containing vesicles to the plasma membrane where they dock and fuse, resulting in increased cell surface GLUT4 levels. Recent work identified a potential further regulatory step, in which insulin increases the dispersal of GLUT4 in the plasma membrane away from the sites of vesicle fusion. EFR3 is a scaffold protein that facilitates localization of phosphatidylinositol 4-kinase type IIIα to the cell surface. Here we show that knockdown of EFR3 or phosphatidylinositol 4-kinase type IIIα impairs insulin-stimulated glucose transport in adipocytes. Using direct stochastic reconstruction microscopy, we also show that EFR3 knockdown impairs insulin stimulated GLUT4 dispersal in the plasma membrane. We propose that EFR3 plays a previously unidentified role in controlling insulin-stimulated glucose transport by facilitating dispersal of GLUT4 within the plasma membrane.

## Introduction

Insulin stimulates glucose transport in adipocytes and muscle. This is mediated by the regulated delivery of the glucose transporter GLUT4 from intracellular stores to the plasma membrane (PM). A wealth of information has underpinned our understanding of intracellular GLUT4 trafficking and the mechanism by which GLUT4-containing vesicles fuse with the PM [1–5].

It has become clear that organization of GLUT4 within the PM is also subject to regulation by insulin; in murine 3T3-L1 adipocytes, GLUT4 in the PM is found in both relatively stationary clusters and as freely diffusible monomers [6]. In the absence of insulin, GLUT4 is retained in clusters at the site of fusion; these clusters nucleate clathrin assembly and are thought to represent sites of GLUT4 internalization. Insulin stimulation was observed to mediate increased delivery of GLUT4 to the PM accompanied by increased dispersal of GLUT4. Two distinct types of GLUT4-vesicle exocytosis were identified: ‘fusion-with-release’ events dispersed GLUT4 within the PM whereas ‘fusion-with-retention’ events retained GLUT4 molecules at the fusion site. In the basal state ∼95% of events observed were ‘fusion-with-retention’ and insulin led to a 60-fold increase in ‘fusion-with-release’ events in 2–3 min [6]. A follow-up study investigated GLUT4 cluster retention and the molecular dynamics guiding GLUT4 exchange in the PM of primary rat adipocytes, and insulin's effects on GLUT4 organization at high resolution [7]. Isolated rat adipocytes were transfected with photo switchable HA-GLUT4-EOS and live cell single molecule tracking was performed with fluorescence photoactivation localization microscopy. The data suggest that insulin had three separable effects that contribute to the shift of GLUT4 molecules within the PM from a clustered to a more dispersed state. Firstly insulin shifted the fraction of dispersed GLUT4 upon delivery to the PM, secondly insulin increased dissociation of GLUT4 monomers from clusters, and lastly insulin decreased the rate of GLUT4 endocytosis [7].

The concept of insulin-stimulated GLUT4 dispersal has been further supported using other imaging methods [7,8]. These approaches confirmed a shift in the distribution of the spatial organization of GLUT4 towards a more dispersed state following insulin stimulation in 3T3-L1 adipocytes. Interestingly, insulin resistance was found to induce a more clustered distribution of GLUT4, with more molecules per cluster, implying that the regulation of post-fusion GLUT4 distribution may be impaired in disease [8]. Such studies underscore a need to identify mechanisms that regulate GLUT4 spatial distribution. Here, we report the identification of one such potential mechanism.

EFR3 was identified from a screen for regulators of GLUT function when heterologously expressed in yeast [9]. EFR3 is a palmitoylated protein responsible for PM localisation of phosphatidylinositol 4-kinase type IIIα (PI4K-IIIα) [10–13]. Here, we reveal that EFR3 is a key regulator of GLUT4 dispersal in the PM. We show that both EFR3 and PI4K-IIIα are required for insulin-stimulated glucose transport. Using direct stochastic reconstruction microscopy (dSTORM), we demonstrate that knockdown of EFR3 reduces insulin-mediated dispersal of GLUT4 and present a model in which EFR3 serves as a regulatory hub controlling insulin-stimulated GLUT4 dispersal in the PM.

## Results

### EFR3 is implicated in GLUT4 trafficking

When heterologously expressed in the yeast *Saccharomyces cerevisiae* the mammalian glucose transporter GLUT4 is retained intracellularly, underscoring the conservation of molecular machinery required for regulated trafficking [14]. Even when localized to the cell surface, GLUT4 does not efficiently transport glucose suggesting that regulation of GLUT4 within the PM might be important [9,15]. We carried out a genetic screen to select mutations in the yeast genome that enable mammalian glucose transporters to support uptake of glucose into yeast cells lacking their own endogenous hexose transporters [9]. Expression of GLUT4 was unable to support growth on glucose of yeast lacking endogenous hexose transporters unless they also carry the recessive mutant *fgy1-1* allele [9]. *fgy1-1* is a mutant allele of *EFR3* (Wieczorke and Boles, personal communication; [16]).

*EFR3* is required for Stt4-containing phosphoinositide kinase patch assembly at the PM [10]. Stt4 is the yeast orthologue of the human type III phosphatidylinositol-4-kinase IIIα which localizes to punctate dots on the PM [12]. Efr3 is similarly found at the cell surface [12,17,18]. This localization requires the addition of palmitoyl moieties to one or more cysteine residues towards the N-terminus. Intriguingly, the *Drosophila melanogaster* Efr3 orthologue Rolling-Blackout is required for synaptic vesicle exocytosis [13], although its precise role is yet to be defined.

These data suggested to us that EFR3 and PI4K-IIIα may play a role in the regulation of glucose transport in response to insulin and prompted us to interrogate the role of EFR3 in insulin regulated GLUT4 trafficking.

### EFR3A is the major isoform expressed in adipocytes

**Figure 1:**
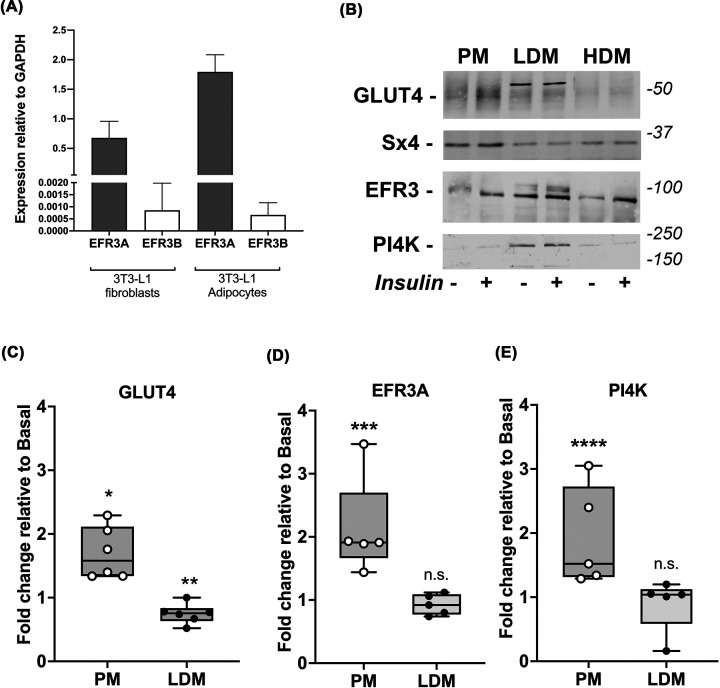
EFR3A is the major isoform in 3T3-L1 adipocytes and is localized to the plasma membrane (**A**) shows RT-PCR analysis of the expression of EFR3A and EFR3B normalized to GAPDH in 3T3-L1 fibroblasts and adipocytes. Data presented is from triplicate biological repeats each with at least three technical replicates (mean and S.D.) EFR3A is the predominant isoform in 3T3-L1 fibroblasts and adipocytes. (**B**) shows a subcellular analysis of 3T3-L1 adipocytes treated with or without 100 nM insulin for 20 min and separated into PM (PM)-enriched, high density microsomes (HDM) and low density microsomes (LDM), which were then immunoblotted for the proteins indicated; figures at right are approximate positions of MW markers in kDa. In each case, 30 μg of protein was loaded in each fraction. Data from a representative experiment is shown, replicated four times with qualitatively similar results. (**C–E**) shows quantification of GLUT4 (C), EFR3 (D) and PI4K-IIIα (E) signals in PM and LDM fractions in the four fraction experiments. GLUT4 levels in the PM increase 1.7-fold (C, *P=0.002) and decrease in the LDM by 25% (**P=0.003) consistent with similar studies. EFR3 and PI4K-IIIα levels in the PM-enriched fractions increase in response to insulin (D, 2.1-fold, ***P=0.012 and E, 1.9-fold, ****P=0.03, respectively). Modest decreases in the LDM fraction for these proteins do not reach statistical significance (n.s.). Syntaxin4 (Sx4) is used as a marker for a protein known to be enriched in the PM [55,56].

There are two EFR3 paralogs in higher eukaryotes, EFR3A and EFR3B. We used RT-PCR to ascertain which are expressed in our experimental model 3T3-L1 cells. Figure 1A shows that EFR3A is highly expressed in 3T3-L1 fibroblasts and adipocytes; levels of EFR3B are 30- to 100-fold lower. EFR3 functions as a part of a protein complex that delivers active PI4K-IIIα to the PM; EFR3 associates with the PM via palmitoylation [10,19]. We therefore asked whether EFR3 and PI4K-IIIα were PM localized in 3T3-L1 adipocytes, and whether insulin modulated this distribution. Subcellular fractionation revealed that, as in yeast [12,18,20], both EFR3 and PI4K-IIIα are found in PM-enriched fractions and within intracellular fractions (Figure 1B). Insulin stimulated an increase in PM-levels of GLUT4 (1.7-fold, *P*=0.002), EFR3 (2.1-fold, *P*=0.012) and PI4K-IIIα (1.9-fold, *P*=0.03) increased in the PM-enriched fractions (Figure 1C–E). The antibodies used to detect EFR3 cannot distinguish between EFR3A and B but given the RT-PCR data in Figure 1A we contend this is likely to represent EFR3A.

### EFR3 and PI4K are required for insulin-stimulated glucose transport in adipocytes

**Figure 2:**
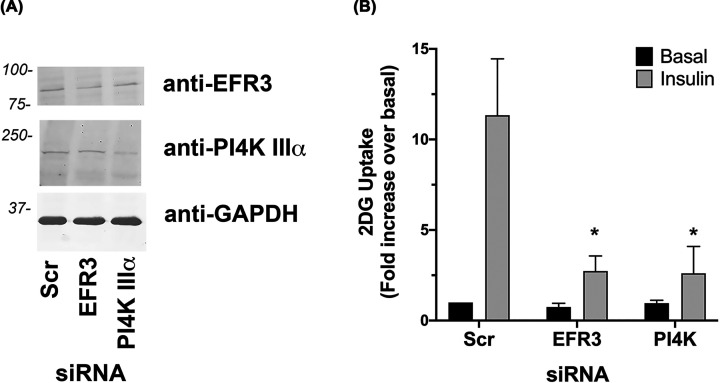
Knockdown of EFR3 impairs insulin-stimulated 2DG transport (**A**) 3T3-L1 adipocytes were electroporated with siRNA designed to knockdown EFR3A or PI4K-IIIα, or scrambled control siRNA (Scr) at day 6 post-differentation as described. Cells were incubated in serum-free media for 2 h, with or without 100 nM insulin for the last 30 min then 2DG uptake measured. Because cell numbers varied considerably between experiments, the data are normalized to the rate obtained in the Scr-cells in the absence of insulin. Data shown as mean and SEM of data from at least three biological repeats, each with five technical replicates. Knockdown of either EFR3 or PI4K-IIIα impaired insulin-stimulated 2DG uptake (*P<0.001 for both). Differences in basal transport rate were not significant. (**B**) shows a representative immunoblot in which levels of EFR3 and PI4K-IIIα, together with GAPDH, are shown in lysates of cells treated with the indicated siRNA. Data from a representative experiment is shown.

To directly assay effects on insulin-stimulated glucose transport we used siRNA to knockdown EFR3 or PI4K-IIIα in 3T3-L1 adipocytes at day 6 post-differentiation. Immunoblot analysis revealed a reduction in EFR3 levels of 48.2% and PI4K-IIIα of 41.5% in cells electroporated with the siRNA against EFR3 and PI4K-IIIα respectively, compared to scr-siRNA treated cells (Figure 2A). Insulin-stimulated a 11.3 ± 3-fold increase in 2-deoxy-D-glucose uptake in cells electroporated with scr siRNA (Figure 2B). Knockdown of EFR3 or PI4K-IIIα reduced this to 2.7 ± 0.8-fold and 2.6 ± 1.5-fold, respectively (Figure 2B).

These observations, together with the localization of EFR3 to the PM prompted us to test the hypothesis that EFR3 knockdown impairs insulin-stimulated glucose transport by an effect on GLUT4 dispersal.

### EFR3 knockdown impairs insulin stimulated GLUT4 dispersal in the PM

Super-resolution imaging approaches have revealed reorganization of GLUT4 in the PM at the single molecule level [6–8]. GLUT4 distribution in the PM was non-homogenous: GLUT4 molecules form clusters or monomers in 3T3-L1 adipocytes. Ripley’s K function spatial analysis revealed that GLUT4 molecules were less clustered after insulin stimulation [8]. We used a similar dSTORM approach to assess the effects of EFR3 knockdown on GLUT4 distribution in the presence and absence of insulin on samples processed using a standard immunofluorescence protocol with a commercially obtained HA-antibody. While this approach generates consistent imaging results the application of fluorescently labelled antibodies decreases the quantitative accuracy of localization of molecules due to their size, a phenomenon known as antibody linkage error. To control for this, we performed comprehensive spatial point pattern analysis rather than quantifying individual cluster descriptors.

HA-GLUT4-GFP expressing 3T3-L1 adipocytes were electroporated with scr-siRNA or siRNA targeting EFR3, incubated with or without 100 nM insulin for 20 min and a dSTORM dataset was collected from cells stained with anti-HA in the absence of detergent, so that only cell surface GLUT4 was immuno-labelled. ThunderSTORM was used to localize each recorded emission of the individual GLUT4 single molecules and generate output files that contain the complete *x/y* coordinates of all molecule localizations from the numbers of cells reported in the figure legends [21].

**Figure 3:**
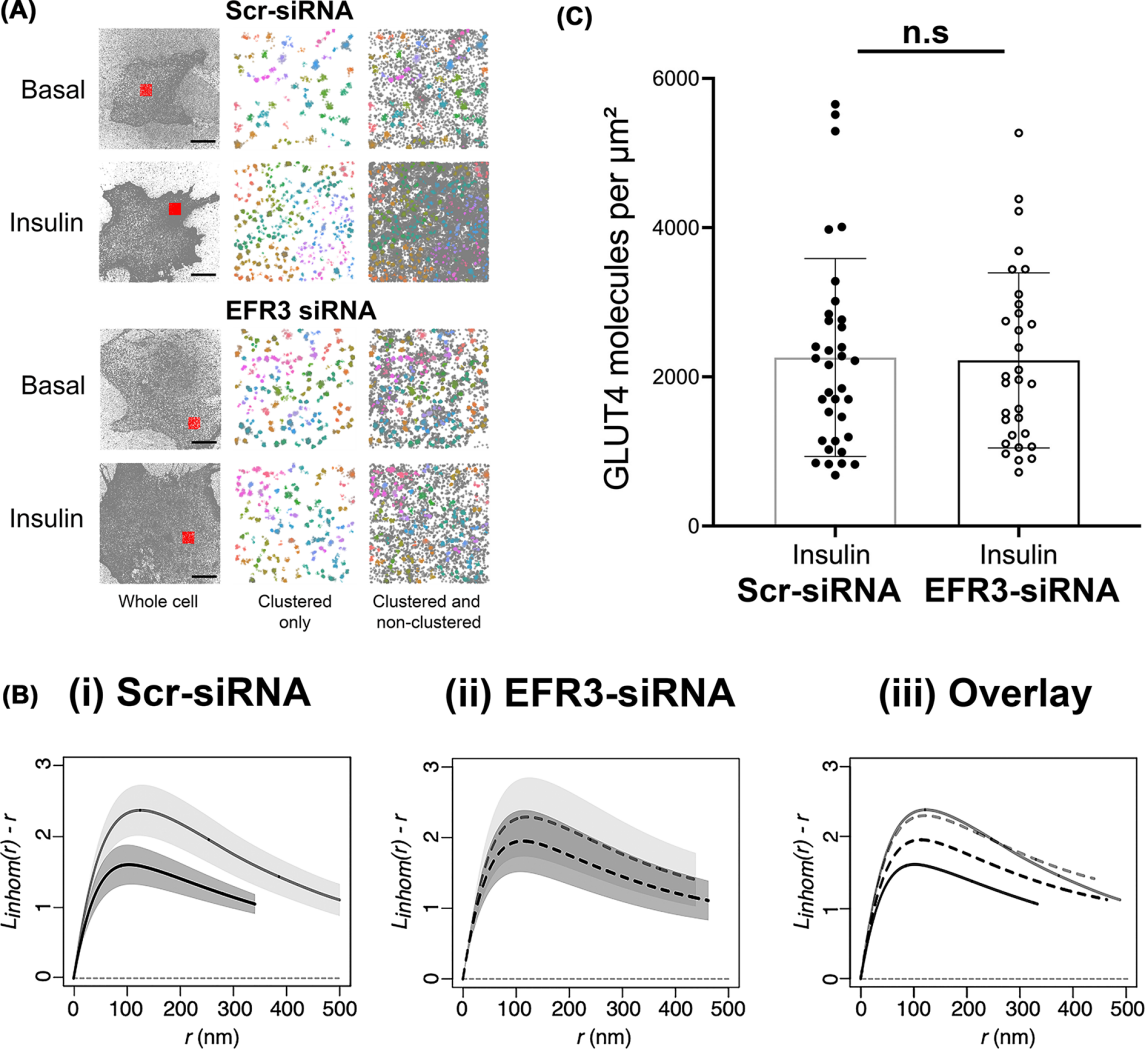
Insulin-stimulated GLUT4 dispersal is impaired upon EFR3 knockdown 3T3-L1 adipocytes stably expressing HA-GLUT4-GFP were electroporated with siRNA to knockdown EFR3, or a corresponding Scr-siRNA at day 6 post-differentiation as described. Cells were stimulated with 100 nM insulin for 20 min, or left untreated (Basal), before fixation and staining for surface HA as decribed in Materials and Methods. dSTORM images were acquired and reconstructions calculated using ThunderSTORM. (**A**) Hierarchical density-based spatial clustering of applications with noise analysis for representative control and EFR3 knockdown basal and insulin-stimulated 3T3-L1 adipocytes. GLUT4 molecule coordinates were processed using a python *HDBSCAN* script written in house. Grey dots indicate single molecule localizations for representative whole cells (left panels). Red boxes indicate region of interests shown in the middle and right panels. The middle panels show coloured molecule clusters identified by *HDBSCAN* within an ROI (‘clustered only’) and coloured clusters plus grey non-clustered molecules identified by *HDSCAN* within an ROI (‘clustered and non-clustered’, right panels) are shown. The representative 4 μm × 4 μm ROI is highlighted by the red square. Scale bars = 10 μm. (**B**) L function analysis of GLUT4 molecule clustering in EFR3 knockdown basal and insulin-stimulated 3T3-L1 adipocytes***.*** GLUT4 molecule coordinates were obtained using ThunderSTORM and its spatial pattern was analysed. We analysed the correlation of points using the variance stabilised L function, which is the transformed version of Ripley’s K function. The L function describes how many points (given by L(r)) can be found within a distance r of any arbitrary point. Empirical estimates of the centred L(r) function are shown in panels labelled (i) control basal and insulin-stimulated cells, (ii) EFR3 knockdown basal and insulin-stimulated cells, (iii) all experimental groups combined. The experiment was carried out independently 4 times on *n*=10 cells per group. Empirical estimates of the L function from each were pooled together to provide a weighted mean and the 83% (or 1.37σ) confidence interval for each group – see text for details. Complete spatial randomness, modelled from a Poisson process, is indicated by the dashed line. (**C**) GLUT4 surface density upon EFR3 knockdown*.* HA (surface GLUT4) localization density was determined from all cells analysed in the presence of insulin. The GLUT4 localisation values do not differ between Scr- and EFR3-siRNA treated groups (n.s.).

We first qualitatively analysed the data using Hierarchical Density-Based Spatial Clustering of Applications with Noise [22,23] to identify clusters of GLUT4 in dSTORM images (Figure 3A). In basal cells *HDBSCAN* identified both GLUT4 clusters (coloured structures; Figure 3A) and dispersed GLUT4 molecules (grey dots; Figure 3A). After insulin-stimulation more GLUT4 clusters are visible in the PM and the density of dispersed GLUT4 molecules is increased (Figure 3A). This result is in line with the current literature stating that approximately 50% of GLUT4 molecules are clustered in the basal state and insulin stimulation induces an increase in the number of GLUT4 clusters in the PM [6,8]. Knockdown of EFR3 had little effect on the numbers of GLUT4 clusters, but insulin-stimulation did not increase the density of GLUT4 monomers, suggesting that GLUT4 dispersal is impaired.

To verify this, we used statistical analysis of spatial point pattern data in R (*spatstat*) to compute an estimate for the edge-corrected inhomogeneous Ripley’s L-function. We first confirmed using null hypothesis testing that all the GLUT4 spatial points were significantly different from complete spatial randomness (indicated by the dashed line in Figure 3B). As shown in Figure 3B, the inhomogeneous L(r) peaks at higher values for basal cells indicating more GLUT4 is found clustered at closer distances compared to insulin-stimulated cells, and closely resembles that reported by others [8]. Knockdown of EFR3 was found to have little effect on the basal distribution of GLUT4, but significantly reduced the dispersal observed in response to insulin (Figure 3B).

We further quantified HA (surface GLUT4) levels from ThunderSTORM datasets [24]. Strikingly, Figure 3C indicates that the level of GLUT4 at the surface in the presence of insulin is not changed by EFR3 knockdown.

**Figure 4:**
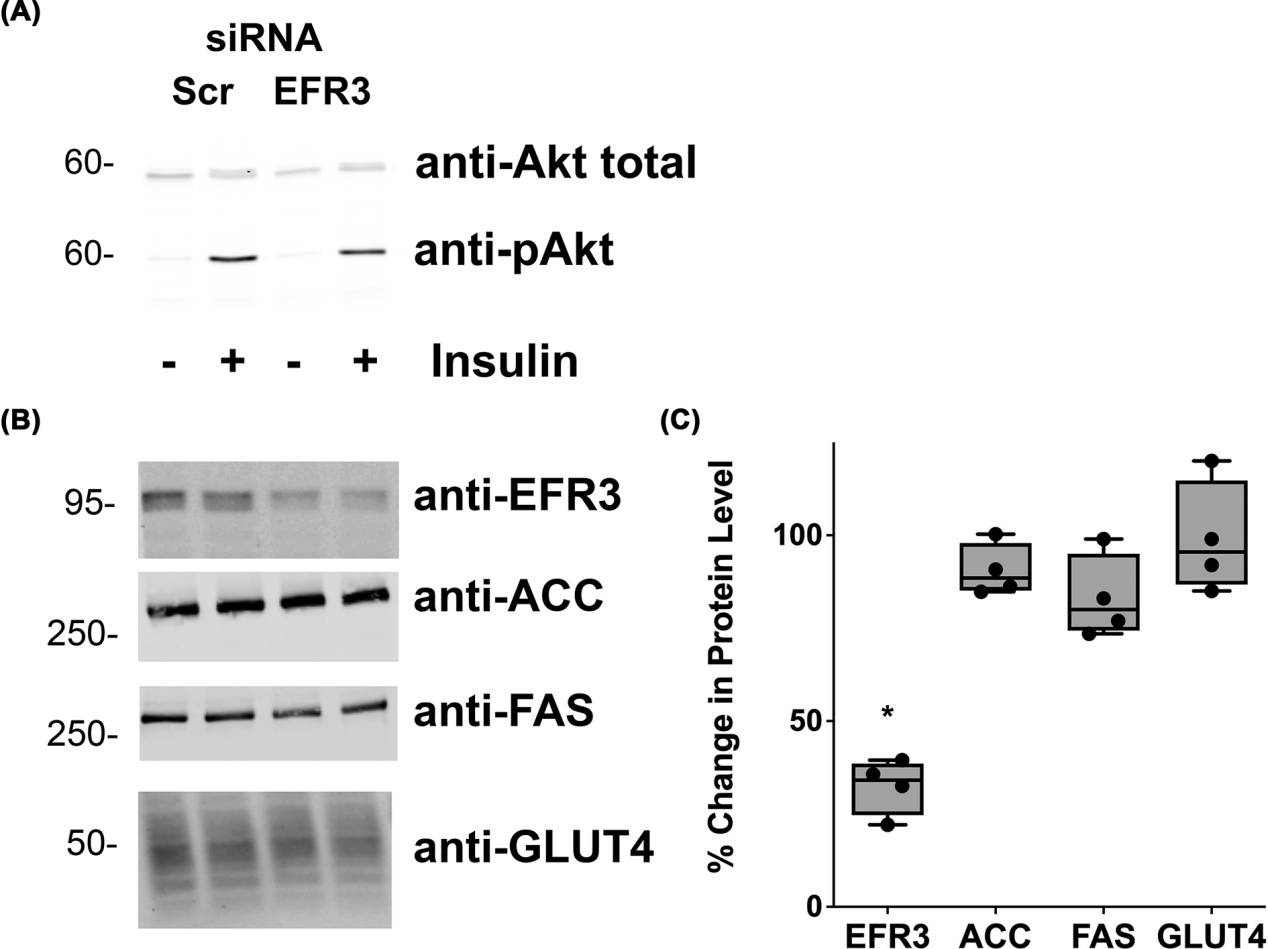
EFR3 knockdown does not impair insulin action or adipocyte differentiation (**A**) 3T3-L1 adipocytes were treated with siRNA designed to knockdown EFR3A or scrambled control siRNA (Scr) at day 6 post-differentiation as described. Cells were incubated in serum-free media for 2 h and 100 nM insulin added for the final 15 min. Cell lysates were prepared and immunoblotted for total Akt or phospho-Akt as shown. Data from a representative experiment are presented. (**B**) Lysates prepared in a similar manner were immunoblotted for EFR3, acetyl coenzyme A carboxylase (ACC), fatty acid synthase (FAS) or GLUT4; representative immunoblots are presented. Data from a typical experiment is shown, with quantification from four experiments of this type presented in (**C**) Data shown as a % change comparing unstimulated samples; *statistically significant decrease, *P*=0.035. Similar data are obtained when the insulin-stimulated lysates are quantified (not shown). ACC, FAS and GLUT4 levels were not affected by EFR3 depletion.

To rule out any effects of EFR3 depletion on insulin signalling of adipocyte differentiation, we replicated EFR3 knockdown in wild-type 3T3-L1 adipocytes and examined the ability of insulin to stimulate signalling processes and screend for effects on adipocyte differentiation markers (Figure 4). It did not change the ability of the cells to respond to insulin as evidenced by a robust increase in Akt phosphorylation in response to insulin (Figure 4A) or change levels of adipocyte markers acetylcoenzyme A carboxylase, fatty acid synthase or GLUT4 itself (Figure 4B,C). Hence the effects on GLUT4 dispersal (Figure 3) and 2-deoxy-D-glucose uptake (Figure 2) are unlikely to be a consequence of either de-differentiation of the cells or a loss of insulin signalling.

These data are therefore consistent with the idea that depletion of EFR3 has impaired the transition from clustered to dispersed GLUT4 in the PM, independent of any effects on GLUT4 levels or insulin signalling/adipocyte differentiation.

## Discussion

While many of the details regarding the intracellular trafficking of GLUT4 are becoming clearer, much less known about the behaviour of GLUT4 once at the PM. Several lines of investigation suggest that controlling GLUT4 at the PM could be a key facet of insulin action. Koumanov et al. developed a cell-free system to study intracellular GLUT4 vesicle fusion with vesicles prepared from the PM of adipocytes; this fusion was cytosol and energy-dependent. Remarkably GLUT4 vesicle fusion with PM-derived vesicles was enhanced 8-fold when PMs were prepared from insulin-stimulated cells, even if internal GLUT4 vesicles and the cytosol were from unstimulated (basal) cells [25]. This study provides compelling evidence for an insulin-regulated event at the PM.

Recent work has suggested that GLUT4 clustering may be an important control mechanism. In murine 3T3-L1 adipocytes, GLUT4 is thought to function as a monomer in the PM, distinct from other transporters such as GLUT1 which are proposed to form oligomers to regulate functional activity [26,27]. Many studies have argued that GLUT4 is non-randomly distributed within the PM of adipocytes (both primary and 3T3-L1 adipocytes) and muscle, with studies describing a punctate distribution at the cell surface [28–30]. Some attribute this to clustering with clathrin or caveolae [31], but recent studies argue against this [6]. These studies have recently been extended by imaging approaches which describe GLUT4 clusters in the PM and the ability of insulin to promote GLUT4 dispersal from these clusters [6–8]. The observation that insulin-stimulated GLUT4 dispersal is reduced in insulin resistant models [8] and the behaviour of GLUT4 vesicles adjacent to the PM is modulated in insulin resistance further emphasises the potential importance of PM-associated GLUT4 events [32]. However, thus far mechanistic detail of how GLUT4 clustering might be modulated has not been forthcoming. Studies in yeast expressing GLUT4 described above focussed our attention on EFR3 and its associated protein PI4K-IIIα.

### EFR3 regulates insulin-stimulated glucose transport

Several lines of evidence presented here argue in favour of an important role for EFR3 and PI4K-IIIα in insulin action. First, levels of EFR3 increase during differentiation of 3T3-L1 adipocytes, a property shared by many of the key molecules involved in insulin action (Figure 1A) [5,33,34]. Furthermore, we show that EFR3 and PI4K-IIIα are present within subcellular fractions enriched for PM markers, and that like GLUT4 both EFR3 and PI4K-IIIα exhibit insulin-dependent increases at the cell surface (Figure 1B,C). Finally, our observation that knockdown of either EFR3 or PI4K-IIIα impaired insulin-stimulated glucose transport provides compelling support for a role for EFR3/PI4K-IIIα in the regulation of GLUT4 (Figure 2). Rescue experiments have not been attempted due to the difficulties of performing such experiments in terminally differentiated 3T3-L1 adipocytes. Nonetheless, it is important to note that knockdown of either EFR3 or PI4K-IIIα impaired insulin-stimulated glucose transport to a similar degree, a result unlikely to be a result of off-target effects and in marked contrast to data obtained using Scr-control siRNAs.

### EFR3 regulates GLUT4 dispersal

Our data support the hypothesis that EFR3 may act as a regulator of GLUT4 dispersal. Using dSTORM we examined the distribution of GLUT4 clusters in the PM and used spatial point pattern data in R to compute an estimate for the edge-corrected inhomogeneous Ripley’s L-function. These data reveal that EFR3 knockdown significantly impairs the ability of insulin to promote dispersal of GLUT4 in the PM (Figure 3B). In this analysis, we used anti-HA to label cell surface GLUT4 followed by detection with a labelled secondary antibody. At high resolution, antibodies can significantly limit how well the image reflects the actual structure by increasing the apparent size of visualized structures; for this reason, we have not attempted to quantify the number of molecules per cluster or cluster density. However a visual representation of the data generated using Hierarchical Density-Based Spatial Clustering of Applications with Noise (Figure 3A) is consistent with insulin driving an increase in the number of clusters and an increase in the density of GLUT4 monomers, similar to that reported by others using this approach [8]. Strikingly, this analysis also clearly reveals that GLUT4 dispersal is impaired upon EFR3 knockdown (Figure 3A,B). To our knowledge, this is the first clue to the mechanism of insulin stimulated GLUT4 dispersal. Quantification of the localisation density of GLUT4 in the PM revealed that the ability of insulin to stimulate GLUT4 translocation was indistinguishable under control or EFR3-knockdown conditions (Figure 3C), and that levels of total GLUT4 were unchanged by EFR3 knockdown (Figure 4B,C). These observations suggest that the insulin-stimulated translocation of GLUT4 to the PM is not significantly impaired in EFR3-depleted cells, but the inability of GLUT4 to undergo insulin-stimulated dispersal underscores the impairment in insulin-stimulated glucose transport (Figure 2). It will be of interest in future studies to ascertain whether similar effects are observed upon knockdown of PI4K-IIIα and to consider whether the observed effects on GLUT4 dispersal are specific for this transporter or are more widely evident for other PM localised proteins.

### Summary

The idea of localization controlling the function and activity of PM resident proteins is well-established [35–40]. Our work provides a link between EFR3 (and PI4K-IIIα) and insulin-stimulated glucose transport, establishing EFR3 as a key locus of insulin action. Analysis of GLUT4 distribution in the PM demonstrates that the insulin-stimulated dispersal of GLUT4 in the PM is dependent upon EFR3. A challenge for the future is to understand how this relates to GLUT4 dynamics in the PM, and how the insulin signalling machinery integrates with the EFR3 scaffold.

## Materials and methods

### Antibodies

Anti-Syntaxin 4 was from Synaptic Systems (Germany; #235003 and 110042, RRID:AB_887853, respectively). Anti-GAPDH [#AM4300. RRID:AB_2536381], anti-GLUT4 [# PA1-1065, RRID:AB_2191454], anti-EFR3A [#PA5-54694, RRID_AB:2640925] and anti-PI4K [#PA5-28570, RRID:AB-2546046] were from ThermoFisher (Renfrew, U.K.). Anti-EFR3 was from Sigma (Dorset U.K.; #HPA023092) and anti-PI4K-IIIα was from AbCam (Cambridge, U.K.; #111565). Anti-HA was from Covance (Suffolk, U.K.; mouse monoclonal MMS101P, RRID:AB_2314672) or from Roche (Wellwyn Garden City, U.K.; RRID:AB_2687407). The Alexa Fluor 647-conjugated anti-HA antibody for dSTORM experiments was from Invitrogen (Renfrew, U.K.; mouse monoclonal #26183-A647, RRID:AB_2610626). Secondary antibodies were from LICOR Biosciences (Cambridge U.K.; donkey anti-rabbit: #925 68023, RRID:AB_2814907; goat anti-mouse #925 68070, RRID:AB_2651128; goat anti-mouse #926 32210, RRID:AB_621842 and goat anti-rat [for FACS] #A-21247, RRID:AB_141778).

### Plasmids

EFR3A- and EFR3B tagged with mCherry, and the corresponding tomato-tagged C(6-9)S mutant were provided by Pietro De Camilli (Yale University) and are described in [12,18].

### Growth and transfection and transport assays of 3T3-L1 adipocytes

3T3-L1 murine adipocytes were purchased from ATCC (via LGC Standards, U.S.A. RRID:CVCL_0123) and grown and differentiated as outlined [34,41]. Stable lines of 3T3-L1 fibroblasts expressing HA-GLUT4-GFP had previously been generated in the lab [42]. Cells were incubated in a 10% CO_2_ humid atmosphere incubator at 37°C.

For electroporation, adipocytes were used at day 6 post-differentiation. Cells were washed in PBS before detaching using 0.05% (w/v) trypsin: 2 mg/ml collagenase. Once detached, cells were washed and transferred to 0.2 cm BioRad Gene Pulser® electroporation cuvette containing 3 nmol Silencer® select pre-designed siRNA and electroporated using settings of 0.18 kV and 975 μF. Cells were then plated and assayed 72 h after electroporation [43]. siRNA against EFR3 was siRNA ID s94606 (ThermoFisher) and PI4K-IIIα siRNA ID s104706 (ThermoFisher).

2-Deoxy-**D**-glucose (2DG) uptake was assayed as in [34]. Non-specific association of radioactivity with the cells were quantified by performing parallel assays in the presence of 10 μM cytochalasin B [44].

### Semi-quantitative reverse transcriptase PCR

mRNA was extracted from the cells in question (see figure legend) using QiaGEN mRNA easy kit as per the manufacturer’s instructions and quantified using a Nanodrop 1000. cDNA was prepared using Applied Biosystems reagents for RT-PCR, and then used in an Applied Biosystems Power SYBR-green PCR master mix. Primers used were (written 5′ to 3′): EFR3A ggtgacagatgaagatcgcc and cacatcatcagaaggcaca; EFR3B aagcccgttcttatccacct and gctgcggctgaattgagta; GAPDH gttgtctcctgcgacttca and ggtggtccagggtttctta.

### Subcellular fraction and immunoblotting

About 10 cm plates of 3T3-L1 adipocytes between day 8 and 10 were serum starved using serum-free DMEM for 2 h and incubated with or without insulin as in the figure legends. Plates were washed using sterile HES buffer, scraped and homogenised using a Dounce homogeniser and subcellular fraction performed as described [34]. This procedure generates a fraction enriched in the PM (PM), high-density membranes (HDM), low-density membranes (LDM) and soluble protein fraction [45–47]. Insulin results in a redistribution of GLUT4 from the LDM to the PM fraction. SDS-PAGE and immunoblotting for proteins within these fractions was performed as outlined [34]. Quantification of immunoblots was performed using Image Studio Light and data analysed using GraphPad Prism software.

### Cell preparation for Dstorm

Prior to measurement, 3T3-L1 adipocytes expressing HA-GLUT4-GFP were serum starved for 2 h. Cells were then stimulated with 100 nM insulin for 20 min or left untreated. Subsequently, cells were fixed with 4% paraformaldehyde (PFA) in PBS overnight at 4°C. The samples were quenched with 50 mM NH_4_Cl in PBS for 10 min at room temperature, washed with PBS then incubated in blocking solution (2% BSA with 5% goat serum in PBS) for 30 min. Afterward, cells were incubated with a conjugated anti-HA antibody coupled to Alexa Fluor 647 at a concentration of 8 μg/ml in blocking solution for 1 h in the dark. Samples were washed with PBS for 10 min three times on an orbital shaker.

### dSTORM image acquisition and reconstruction

The dSTORM image sequences were acquired on an Olympus IX-81 microscope equipped with Olympus Cell⁁R acquisition software, an ImageEM EM-CCD 512 × 512 camera (Hamamatsu U.K.) and an Olympus × 150 UAPO oil lens with a numerical aperture of 1.45 and a resulting pixel size of 106 nm. Cold dSTORM imaging buffer containing 50 mM mercaptoethylamine (MEA) in PBS (pH 8) was pipetted into cavity slides and coverslips were sealed onto slides using dental paste to avoid oxygen entry. After mounting of samples image sequences of 10,000 frames were acquired in total internal reflection fluorescence (TIRF) configuration using 647 nm laser light at 100% power (150 mW). Images were recorded on an Andor iXon 897 EMCCD camera using a centered 256 × 256 pixel region at 30 ms per frame for 10,000 frames and an electron multiplier gain of 200 and pre-amplifier gain profile 3. The dSTORM data were processed using the freely available ImageJ plugin ThunderSTORM [21]. The image reconstruction parameters chosen are lined out in the following: pre-detection wavelet filter (B-spline, scale 2, order 3), initial detection by local maximum with 8-connected neighbourhoods (radius 1, threshold at two standard deviations of the F1 wavelet), and sub-pixel localization by integrated Gaussian point-spread function (PSF) and maximum likelihood estimator with a fitting radius of 3 pixels. The first pass detected localizations were filtered according to the following criteria: an intensity range of 500–5000 photons, a sigma range of 25–250, and a localization uncertainty of less than 25 nm. Subsequently, the filtered data set was corrected for sample drift using cross-correlation of images from 5 bins at a magnification of 5. Repeated localisations, such as can occur from dye re-blinking, were reduced by merging points which re-appeared within 20 nm and 1 frame of each other.

### HDBSCAN analysis

The density-based spatial clustering of applications with noise (*DBSCAN*) has become one of the most common data clustering algorithms since its development [22]. Density-based clustering defines clusters as areas of higher density compared with the remainder of the data points. Hierarchical *DBSCAN* (*HDBSCAN*) uses an unsupervised learning algorithm to find clusters of varying densities [23]. HDBSCAN identifies regions of the data that are denser than the surrounding space and considers cluster hierarchy which is shaped by multivariate modes of the underlying distribution [23,48]. ROI of 4 μm × 4 μm were selected in ImageJ for each cell and GLUT4 molecule coordinates were clustered using *HDBSCAN* parameters of *min_cluster_size* = 5 and *min-samples* = 30 to provide clear visualisation of the dataset. The *HDBSCAN* package (v0.8) was implemented on Python (v3.6) was downloaded from Github (https://github.com/scikit-learn-contrib/hdbscan) [49].

### Spatial statistics analysis in R

Spatstat is a package for analysing spatial point pattern data featuring a generic algorithm for fitting point process models to point pattern data [50]. Analysis of spatial statistics was carried out using the *spatstat* package (v1.64) for R (v3.2). GLUT4 clustering does not show uniform point density and statistics of point pattern correlation with correction for point pattern inhomogeneity and edge correction was applied. Cell outlines were chosen as ROI for the analysis using ImageJ. dSTORM localisations only within cells were further analysed. Ripley’s K function defines and measures covariance in a point process with clustered patterns showing positive covariance, independently placed points showing zero variance and dispersed points showing negative covariance [51].

The K function of a point process shows the expected number of neighbours of a point of a typical stationary point process **X** found at **u** that is within a distance ≤ **r**:

$$K(r)=\;\frac{1}{\lambda }E[t(u,r,X)|u\;\varepsilon X]$$

The empirical Ripley’s K function is obtained by measuring all the pairwise distances of points within one cell then standardized for the density of points.

The variance stabilized L function transforms K(r) into a straight line and is defined as:

$$L(r)=\sqrt{\frac{K(r)}{pi}}$$

The empirical L(r) was calculated for each cell and pooled. For visualisation purposes, L(r) was centered, which is shown in the relevant figures. The experiment was carried out independently four times for a total of *N*=10 cells per group. Empirical estimates of the L function from each were pooled together to provide a weighted mean and the 83% (or 1.37σ) confidence interval for each group. Statistical differences between group means were assessed by overlap of 83% confidence intervals. In statistics checking for overlap of 83% confidence levels has been frequently reported as a method to assess whether two means are significantly different from each other or not at the α = 0.05 level [52–54].

### Localization density of GLUT4 molecules

Localization density of GLUT4 molecules in the PM was assessed using the ImageJ plugin LocFileVisualizer_v1.1 as described [24].

## Data Availability

Datasets are available from the corresponding author. Raw immunoblots are provided to the journal. This is also stated on page 13 of the manuscript file.

## Open Access

Open access for this article was enabled by the participation of University of Strathclyde in an all-inclusive *Read & Publish* agreement with Portland Press and the Biochemical Society under a transformative agreement with JISC.

## Abbreviations

BSA: bovine serum albumin
dSTORM: direct stochastic optical reconstruction microscopy
2DG: 2-deoxy-D-glucose
GAPDH: glyceraldehyde-3-phosphate dedhydrogenase
GLUT4: glucose transporter 4
HDM: high-density membranes
LDM: low-density membranes
MEA: mercaptoethylamine
PBS: phosphate buffered saline
PFA: para-formaldehyde
PI4K-IIIα: phosphatidylinositol 4-kinase type IIIalpha
PM: plasma membrane
PSF: point-spread function
ROI: region of interest
RT-PCR: reverse transcription PCR
SDS-PAGE: sodium dodecyl sulphate polyacrylamide gel electrophoresis
TIRF: total internal reflection
